# Female-specific effects of the catechol-O-methyl transferase Val^158^Met gene polymorphism on working memory-related brain function

**DOI:** 10.18632/aging.104059

**Published:** 2020-11-22

**Authors:** Jialing Fan, Caishui Yang, Zhen Liu, He Li, Yan Han, Kewei Chen, Chuansheng Chen, Jun Wang, Zhanjun Zhang

**Affiliations:** 1 State Key Laboratory of Cognitive Neuroscience and Learning, Beijing Normal University, Beijing 100875, China; 2 National Institute on Drug Dependence, Peking University, Beijing 100191, China; 3 Institute of Basic Research in Clinical Medicine, China Academy of Chinese Medical Sciences, Beijing 100700, China; 4 Department of Neurology, Yueyang Hospital of Integrated Traditional Chinese and Western Medicine, Shanghai University of Traditional Chinese Medicine, Shanghai 200437, China; 5 Banner Alzheimer’s Institute, Phoenix, AZ 85006, USA; 6 Department of Psychological Science, University of California, Irvine, CA 92697, USA; 7 BABRI Centre, Beijing Normal University, Beijing 100875, China

**Keywords:** *COMT*, working memory, fMRI, background functional connectivity, sex

## Abstract

The catechol-O-methyltransferase (*COMT*) Val^158^Met polymorphism has been associated with working memory (WM) in many studies, but the results have not been consistent. One plausible explanation is sex-specific effects of this polymorphism as reported in several studies. The current study aimed to explore the sex-specific effects of the *COMT* Val^158^Met polymorphism on WM-related brain function in an elderly sample. We found that Val homozygotes outperformed Met allele carriers on the backward digit span subtest for both males and females. The triangular part of the left inferior frontal gyrus and the left inferior temporal gyrus exhibited higher activation in Met allele carriers compared with Val homozygotes during the n-back task, while the background functional connectivity (bFC) between the left angular gyrus (ANG) and the right ANG was enhanced in Val homozygotes as compared to Met allele carriers. Finally, the associations between brain activation, bFC (among various regions), and WM performance were identified only in specific genotype groups of the female participants. These findings provide new insights into the role of *COMT* Val^158^Met gene polymorphism in brain function, particularly its female-specific nature.

## INTRODUCTION

The capacity to temporarily store and process information is conceptualized as working memory (WM). It plays a crucial role in all forms of higher cognition and day-to-day functioning [1]. WM has been found to have a moderate level of heritability, with 43% to 49% of its variance attributable to genetic factors [2]. Developmental research has further showed that WM’s heritability is stable across the life span and may become even stronger in older adulthood [3–5]. Of potential candidate genes, the most frequently studied is the catechol-O-methyl transferase (COMT) gene [6, 7] located on chromosome 22q11. One of the main functions of this gene’s protein product, the enzyme COMT, is to regulate the degradation of dopamine (DA) [8, 9]. The best known polymorphism of *COMT* is the Val^158^Met single nucleotide polymorphism (SNP), whose methionine (Met) substitution of a valine (Val) would lead to a four-fold reduction in COMT enzyme activity and hence slower DA degradation [10].

As the cortical expression of DA is regional and might be greatest in the frontal lobe [11], the *COMT* Val^158^Met polymorphism has been thought to be of particular relevance to prefrontal functions such as WM [6]. However, empirical evidence for the impact of the *COMT* genotype on WM performance has been mixed. Some studies showed that Met allele carriers outperformed Val homozygotes in a dose-dependent fashion on WM-related tests (e.g., the Letter and Number Sequencing task, N-back test, and Wisconsin Card Sorting Test) [12–14], but a few other studies [15, 16] and one meta-analysis [17] identified a reversed pattern of the *COMT* effect with the Val allele being linked to better WM performance, and yet another recent meta-analysis [18] reported that this polymorphism had no significant association with WM as well as intelligence in general.

One possible reason for the inconsistent findings across genetic association studies is that the effects of *COMT* genotype are age- and sex-dependent. In terms of age dependency, *COMT*’s effects may be enhanced in old age [19] because the aging brain has limited neural resources, which would magnify genetic effects. The conjecture has been called the resource-modulation hypothesis [3, 4]. In terms of sex dependency, animal experiments found sexually dimorphic changes caused by COMT deficiency [20, 21]. Human studies have also revealed complex interactions between sex and *COMT* genotype, showing sex-specific genotypic effects on a range of cognitive functions, including WM, memory and verbal ability in children and old adults [22–24]. Such sex-*COMT* interactions might have been due to the role of estradiol in regulating COMT activity through its down-regulation gene expression [25]. Considering the sex and age dependency of *COMT*’s effects, the current study used an elderly sample to examine sex differences in *COMT* Val^158^Met polymorphism’s effects on WM and related brain function.

Consistent with the hypothesis that DA expressed in the prefrontal cortex serves as the mechanism underlying the link between *COMT* and WM, previous neuroimaging studies have shown a relationship between the Val allele and greater prefrontal activation during WM tasks in both healthy adults and schizophrenia patients [26–28], although other studies reported that the Met allele was associated with higher fronto-striatal activation during WM-related tasks such as inhibitory control and emotional recognition tasks [29, 30]. In addition to the frontal lobe, other regions (e.g., posterior cingulate cortex) have also been implicated in the association between *COMT* polymorphism and WM [31], which is consistent with the notion that WM relies on distributed regions [32, 33]. Therefore, it is important to examine both regional activities as well as interregional connectivity when studying the role of COMT in WM and related brain function.

In sum, the current study aimed to examine the sex-specific effects of the *COMT* Val^158^Met polymorphism on WM and related brain function in cognitively normal elderly adults. All participants took the digit span test and the imaging subsample performed an n-back WM task while being scanned. Both task-related activation and background functional connectivity (bFC) [34, 35] were analyzed to examine genotypic effects on both local cortical activity and cortical functional coupling.

## RESULTS

### Demographics

The frequencies of *COMT* Val^158^Met genotypes did not deviate from Hardy-Weinberg equilibrium (p > 0.05). In terms of demographic factors for the whole sample, males were older (p < 0.01) and had more years of education (p < 0.01) than were the females (Table 1). For the imaging subsample, male participants were slightly older than the female ones (p = 0.04), and no significant sex difference was found in years of education (Supplementary Table 1). Demographic factors did not differ by genotype or by the interaction between genotype and sex. Finally, the imaging subsample did not differ from the whole sample in terms of the distribution of the four groups (genotype by sex), Wald χ^2^-test (p = 0.34).

**Table 1:** Demographics and working memory performance of all participants.

	**Met allele carriers (N = 289)**		**Val homozygotes (N = 370)**		***COMT* F/χ^2^(p)^a^**	**Sex F/χ^2^(p)^a^**	***COMT* × Sex F/χ^2^(p)^a^**
	**Male (N = 105)**	**Female (N = 184)**	**Male (N = 136)**	**Female (N = 234)**			
Age, yrs	67.37±7.65	64.24±7.01	66.63±7.74	64.04±6.73	0.65(0.42)	23.87(< 0.01)	0.21(0.65)
Education, yrs	11.99±3.22	10.95±3.26	11.78±3.52	11.25±3.09	0.03(0.89)	8.78(< 0.01)	0.89(0.35)
APOE ε4 carriers	22(21%)	27(15%)	16(12%)	34(15%)	1.51(0.22)	0.17(0.68)	4.08(0.25)
MMSE, score	28.28±1.55	28.17±1.49	28.26±1.50	28.03±1.50	0.64(0.42)	2.59(0.11)	0.45(0.50)
DST, score							
Forward	7.72±1.16	7.37±1.37	7.63±1.23	7.44±1.27	0.01(0.76)	6.34(0.01)	0.47(0.49)
Backward	4.43±1.33	4.18±1.25	4.73±1.30	4.43±1.23	6.68(0.01)	6.25(0.01)	0.16(0.69)
2-back task performance ^b^							
Reaction Time, ms	591.95±95.44	619.44±123.27	637.11±107.37	608.85±113.59	0.70(0.40)	0.02(0.89)	1.72(0.19)
Accuracy Rate	0.93±0.05	0.92±0.05	0.92±0.05	0.89±0.10	2.48(0.12)	5.56(0.02)	0.07(0.80)

Values are mean ± SD.

*COMT*, catechol-*O*-methyltransferase Val^158^Met genotype; MMSE, Mini-Mental State Examination; DST, Digit Span Subtest.

^a^Comparisons between groups were performed using Wald χ^2^-test for *APOE* ε4. Two-way analysis of covariance (two-way ANCOVA) was used to determine the main effect of the genotype and sex on the MMSE and working memory performance, as well as the interaction between them (age, education, and *APOE* ε4 as covariates).

^b^2-back task performance was only available for imaging subsample (Met allele carriers-Male: N=19, Met allele carriers-Female: N=25, Val homozygotes-Male: N=31, Val homozygotes-Female: N=46).

### WM performance

For the whole sample, the Val homozygotes outperformed the Met allele carriers on the backward digit span task (p = 0.01), but the two groups did not differ significantly in terms of their performance on the forward digit span task. Males performed better than females on both forward (p = 0.01), and backward digit span tasks (p = 0.01). There was no significant interaction between sex and *COMT* genotype for either of the digit span tasks (all p > 0.05).

For the imaging subsample, no significant main or interaction effects of sex and *COMT* genotype for either of the digit span tasks (all p > 0.05). Males performed better than females on the 2-back WM task in the scanner (p = 0.02). For this sample, there were no significant main effect of genotype or interaction between sex and *COMT* genotype for the 2-back task (p > 0.05).

### Effects of *COMT* genotype and sex on task-related brain activity

Supplementary Figure 1 shows brain activities during the 2-back WM task for each of the four sex-by-genotype groups. Activated regions were mainly clustered in the middle frontal gyrus, inferior frontal gyrus, inferior parietal lobule, and inferior temporal gyrus, with the regions of deactivation mainly located in the middle frontal gyrus, posterior cingulate, insula and middle temporal gyrus.

**Figure 1:**
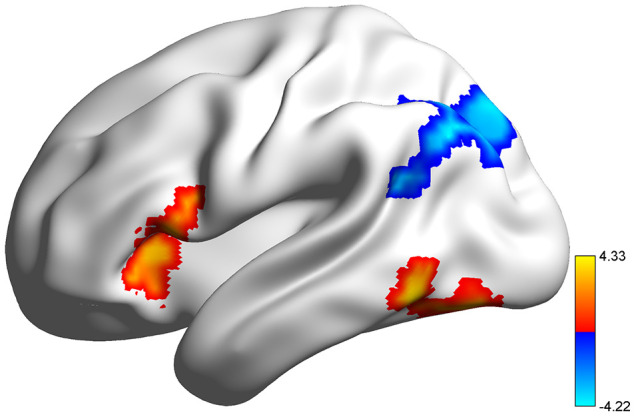
**Rendering of regions with significant *COMT* genotype and sex effects on task-related brain activation.** All results were corrected at voxel p < 0.005 and cluster-level false-positive rate p < 0.05. warm color, *COMT* genotype effect; cool color, sex effect.

Figure 1 and Supplementary Table 2 show the sex and genotype differences in task-related brain activities. Significant *COMT* genotype effects were identified in the left inferior temporal gyrus (ITG) and triangular part of the left inferior frontal gyrus (IFGtri), and the activation was higher in the Met allele carriers than the Val homozygotes. Males showed significantly higher activation in the left angular gyrus (ANG) than did females. There were no significant genotype-by-sex interactions in any region.

We also conducted analyses within regions that were deactivated during the task. There were no main effects of genotype or sex, but two clusters, the right precuneus (PCUN) and the right superior occipital gyrus (SOG), showed significant interactions between genotype and sex, with significantly reduced deactivation in male Met allele carriers than the other three groups (Supplementary Figure 2).

### Association between brain activity and n-back task performance

**Figure 2:**
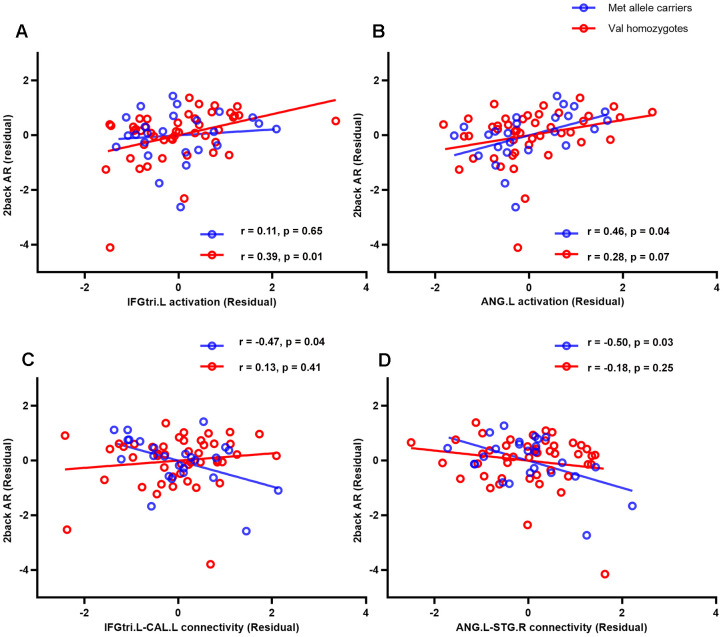
**Correlations between female participants’ performance (accuracy rate) on the 2-back WM task and activations in two brain regions** (the triangular part of left inferior frontal gyrus (**A**) and left angular gyrus (**B**)) and these regions’ background functional connectivity with left calcarine (**C**) and right superior temporal gyrus (**D**), respectively. AR, accurate rate; IFGtri.L, triangular part of left inferior frontal gyrus; ANG.L, left angular gyrus; CAL.L, left calcarine; STG.R, right superior temporal gyrus.

Partial correlation analyses were conducted for each group between significant brain regions and performance (accuracy rate, AR) on the 2-back WM task. As shown in Figure 2, a significant correlation between the activation in the left IFGtri and 2-back AR was found in female Val homozygotes (r = 0.39, p = 0.01), but not for the other three groups. The strength of this association was significantly stronger than that for male Met allele carriers (Z = 1.69, p = 0.05, one-tailed) and male Val homozygotes (Z = 2.19, p = 0.01, one-tailed), but did not differ from that for the female Met allele carriers (Z = 1.15, p = 0.13, one-tailed). Activation in the left ANG was correlated significantly or marginally with 2-back AR mainly for females regardless of their genotype (all females: r = 0.33, p = 0.01; female Met carriers: r = 0.46, p = 0.04; female Val homozygotes: r = 0.28, p = 0.07). The correlation for female Met carriers was significantly higher than that for male Met carriers (Z = 1.83, p = 0.03), but no other group differences were found. Finally, no significant associations were found between activations in the deactivated regions and WM performance.

### Effects of *COMT* genotype and sex on background functional connectivity

Supplementary Figure 3 shows the seed regions (spheres with 6 mm radius) identified based on task-related brain activity analyses and correlations with 2-back WM performance. The seeds were located in the left ANG, the left IFGtri, and the left ITG. We then calculated the bFC between each seed region and the rest of the brain and investigated the effects of the *COMT* genotype and sex on bFC (see Supplementary Table 3 for significant results).

**Figure 3:**
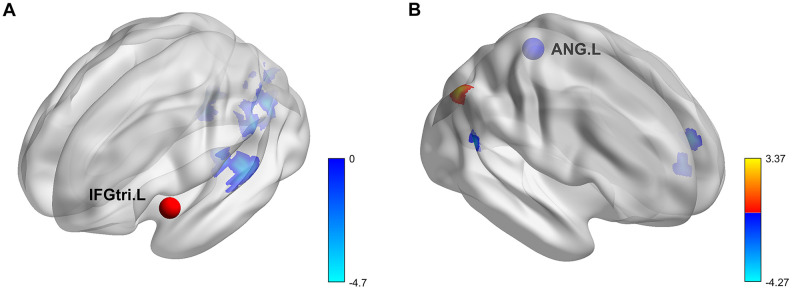
**Rendering of regions with significant *COMT* genotype and sex effects on task-based background functional connectivity.** (**A**) Seed region, IFGtri.L. (**B**) Seed region, ANG.L. All results were corrected at voxel-level p < 0.005 and cluster-level false-positive rate p < 0.05. IFGtri.L, triangular part of left inferior frontal gyrus; ANG.L, left angular gyrus; warm color, *COMT* genotype effect; cool color, sex effect.

For the seed region of the left IFGtri, there were significant sex differences in its connectivity with the following regions: left calcarine (CAL), right CAL, left superior temporal gyrus (STG) and left rolandic operculum (ROL). The connectivity was stronger for females than for males (Figure 3A). No significant genotype effect or sex-by-genotype interactions were found.

For the seed region of the left ANG, there were significant genotype and sex differences in its connectivity with other brain regions (Figure 3B). Specifically, the bFC between the left ANG and right ANG was higher in the Val homozygotes than Met allele carriers. The connectivity between the left ANG and two regions (left medial superior frontal gyrus (SFGmed) and the right superior temporal gyrus (STG)) were lower for males than females. No significant sex-by-genotype interaction was found.

For the seed region of the left ITG, there were no sex or genotype effects in its connectivity with the rest of the brain.

### Association between task-based background functional connectivity and n-back task performance

The bFCs that showed significant sex and/or genotype effects were extracted and correlated with performance on the 2-back WM task using partial correlation analyses. The results showed that the strength of connectivity between left IFGtr and left CAL was negatively correlated with 2-back AR, especially in female Met allele carriers (r = -0.47, p = 0.04, Figure 2C). The correlation was significant lower for female Met allele carriers than female Val homozygotes (Z = -2.47, p < 0.01, one-tailed) and male Val homozygotes (Z = -2.19, p = 0.01, one-tailed), but it did not differ between female and male Met allele carriers (Z = -0.81, p = 0.21, one-tailed).

The connectivity between left ANG and right STG was also negatively associated with 2-back AR (r = -0.50, p= 0.03, Figure 2D). The correlation was significant lower for female Met allele carriers than male Met allele carriers (Z = -1.75, p = 0.04) and male Val homozygotes (Z = -1.85, p = 0.03), but it did not differ between female Met allele carriers and female Val homozygotes (Z = -1.38, p = 0.08).

## DISCUSSION

In this study, we examined the sex-specific effects of the *COMT* Val^158^Met polymorphism on WM and related brain function in a sample of cognitively normal elderly adults. In terms of the *COMT* genotype’s effects on behavioral results, we found only one significant result, with Val homozygotes scoring higher than Met allele carriers on the backward digit span task for the whole sample. There were no other significant genotype effects on WM (i.e., forward digit span for the whole sample, both types of digit span for the imaging subsample, and n-back WM for the imaging subsample). This pattern of limited evidence linking *COMT* genotype to behavioral measures of WM is consistent with the emerging conclusion based on recent meta-analyses [17] and other studies [15, 16].

Although the imaging subsample was much smaller and hence had less statistical power to yield genotype effects, we found significant genotype effects, mostly sex-dependent, on the more proximal measures of WM, namely, task-related brain activity and task-based functional connectivity. In terms of brain activation during performance of the n-back task, the left IFGtri and the left ITG exhibited higher activation in Met allele carriers compared with Val homozygotes. Using positron emission tomography, researchers found that the DA receptors availability in Val homozygotes was higher (lower DA level) across many regions of the brain, suggesting *COMT* gene may affect the function of many regions [36]. And during WM task, DA receptors availability in prefrontal cortex (PFC) [37, 38] and left medial temporal structures [39] decreased during WM task, suggesting a regionally specific frontal-temporal dopaminergic network involved in WM and DA release in these regions might relate to task performance. Also, previous fMRI research has localized *COMT* genotypic effects in PFC [40], as well as more posterior regions including the cingulate cortices, parietal and temporal regions [26, 41–43], but the results have been inconsistent in terms of which allele is associated with increased task activation. Several explanations have been proposed. First, the well-known U-shaped curve of the efficacy of DA signaling (i.e., an optimum amount of DA for the best performance) [44, 45] might have contributed to this inconsistent literature because differences across studies in task difficulty and the experimental context could have placed the studies in different sections of the curve. Second, age range of the samples may also have contributed to the mixed findings because the cortical DA level peaks at puberty and shows a persistent decrease through adulthood [46, 47]. Third, the pattern of results may also differ depending on whether the samples were healthy controls or those with mental disorders such as schizophrenia [17]. Finally, the mixed findings might also have been due to the interactions between sex and genotype. Specifically, previous research has shown the effects of the downregulation of estrogen on the COMT gene and protein [25], leading to 20%-30% less COMT activity in females and thus a different functional level of DA signaling [48].

Our study also found genotype and sex effects on task-based bFC. In terms of sex differences, the bFC between the left IFGtri and four other brain regions (i.e., the left CAL, right CAL, left STG and left ROL) was significantly enhanced in female participants as compared to male participants. In terms of genotype differences, the bFC between the left ANG and the right ANG was enhanced in Val homozygotes as compared to Met allele carriers. To date, only a few studies have examined the genotypic effects on the functional connectivity but they show consistent results regardless whether they examined resting-state or task-related functional connectivity. Resting-state data revealed that the Val allele was linked to higher connectivity among subregions in the frontal lobe (e.g., anterior medial PFC, lateral PFC) and between frontal regions and subcortical structures (e.g., amygdala, hippocampus) [49, 50], and task-related data showed significant FC increases in Val homozygotes, mainly between brain regions involved in the fronto-parietal network [28, 51]. Our results based on the bFC are consistent with this growing literature. The bFC approach retains the simplicity of resting-state connectivity while accounting for different cognitive states. It divided the blood oxygenation level–dependent signal (BOLD) by both the evoked activity in response to the stimulus and the activity related to the maintenance of the current cognitive state [34, 35]. Consequently, the bFC is independent of cortical activation and reflects functional connectivity underpinning specific cognitive processes. With this approach, we found that regions with genotypic effects on activation showed sex differences in terms of functional connectivity and that regions with sex effects on activation showed genotype differences for bFC. These results highlight the important contributions of both regional activity and inter-regional functional coupling when investigating genetic influence on brain functional activity which is sex-specific.

The most interesting finding of our study was the sex-specific effects of *COMT* on the association between bFC and WM performance. We found that the association between bFC (among various regions) and WM performance was stronger in female Met allele carriers than other groups. Besides, the correlations between activation and WM performance we found were also specific to a genotype in female participants. These results are consistent with the growing literature on female-specific effects of *COMT* on cognitive function [22, 52, 53] and brain structure [54–56]. Kempton and colleagues found an interaction between *COMT* genotype and sex on brain activation during an affective processing task, with Val homozygotes showing greater activation in right temporal pole than Met homozygotes in females [57]. Similarly, in their study of brain function during the delay discounting task, Elton et al. found significant sex-*COMT* interactions for a wide range of measures including behavioral performance on the task, task-related activation, rest-state functional connectivity, network attributes such as global and local efficiency, and the functional connectivity flexibility [58]. The effect of *COMT* genotype on brain activation in Velocardiofacial syndrome has been found to be moderated by sex [59]. Taken together these results, the evidence is strong that sex moderates *COMT* genotype effect on a wide range of brain function and structure. Furthermore, the results appeared to be female-specific, suggesting that the *COMT* genotype was more sensitive and regulatable in female participants. This sex-genotype interaction may reflect sexually dimorphic effects due to estrogen’s regulation of COMT activity as mentioned above.

Several limitations of this study should be acknowledged. First, dose effects of the alleles were not specified in the imaging analyses. Due to the limited number of Met homozygotes (n = 4 in the imaging subsample, which did not deviate from Hardy-Weinberg equilibrium), we combined Met homozygotes and heterozygous individuals in the Met allele carrier group. Further studies with larger imaging sample would provide a greater effect size and could address the possible dose effect. Second, the COMT enzyme activity and DA level were not measured in our participants. Such information would have added physiological evidence for our results. Nevertheless, the available evidence suggests that the sex-specific *COMT* genotypic effect is robust. Finally, connectivity results were obtained through seed-based bFC. Future work could explore FC among additional regions and even the whole brain to further depict the *COMT* effects on brain function.

In summary, we observed significant effects of both *COMT* genotype and sex on WM task-related activity and task-based functional connectivity. Some of the genetic effects, especially those on the association between bFC and WM performance were specific to female participants. These findings provide new insights into the role of *COMT* Val^158^Met gene polymorphism in brain function and its female-specific nature. Our findings underscore the importance of integrating sex when examining the genetic effect on cognitive function.

## MATERIALS AND METHODS

### Participants

Participants were enrolled in the Beijing Aging Brain Rejuvenation Initiative (BABRI) [60], which is an ongoing community-based cohort study focusing on cognitive, neuroimaging and sociodemographic factors relevant to aging and dementia. The study was approved by the Institutional Review Board (IRB) of Beijing Normal University, and written informed consent was obtained from each participant.

The inclusion criteria included: a) native Chinese speakers; (b) 50 years of age or older; (c) at least 6 years of education; (d) 24 or higher on the Chinese version of the Mini-Mental State Examination (MMSE) [61]; (e) no history of any neurologic, psychiatric, or systemic illnesses known to influence cognitive function; and (f) no mild cognitive impairment or dementia per current criteria [62, 63].

All 659 participants took the digit span subtest (DST) from the Wechsler Adult Intelligence Scale-Revised [64]. Blood samples were collected from all participants and used for genetic analysis. A subsample of 138 participants was scanned with fMRI three to six months after the initial cognitive assessment.

### Genotyping

*COMT* Val^158^Met was genotyped using TaqMan allele-specific assays on the 7900HT Fast Real-Time PCR System (Applied Biosystems, Foster City, CA). Another two SNPs, rs429358 and rs7412, were also genotyped to determine the *APOE* genotype (ε4 carriers or non-carriers). The sample success rates for all three SNPs were 100% (i.e., no failures across the participants to ‘call’ the polymorphisms), and the reproducibility of all the genotyping was 100% according to a duplication of at least 10% of the genotypes. Based on the *COMT* genotyping, participants were divided into two groups, 289 Met allele carriers and 370 Val homozygotes.

### Experimental paradigm

A block periodic design that incorporated alternating 0-back, 1-back, and 2-back conditions was used during the n-back task in the scanner. Participants were trained prior to scanning to ensure task compliance. During each condition, three blocks of single digits (0-9) were pseudo-randomly shown to the participants. During the 0-back condition, participants were required to press a button when the target digit (e.g., 1) appeared on the screen. During the 1- or 2-back condition, participants pressed a button when the digit shown on the screen matched the one presented one or two items back. There were three blocks for each condition, and every block started with a 10-s cue presentation that indicated 0-, 1- or 2-back, followed by 20 consecutive trials of single-digit stimuli (1000-ms duration and 1000-ms inter-stimulus interval).

The responses and reaction time were recorded by an MRI-compatible response button box. The stimuli were presented using E-Prime version 1.0 software (Psychology Software Tools, Inc., Pittsburgh, PA).

### MRI data acquisition

MRI data were collected on a 3.0 T Siemens Trio scanner at the Imaging Center for Brain Research at Beijing Normal University. The WM task fMRI images were acquired using an echo-planar imaging (EPI) sequence with the following parameters: 33 axial slices, repetition time (TR) = 2000 ms, echo time (TE) = 30 ms, slice thickness = 3.5 mm, flip angle = 90°, field of view (FOV) = 200 × 200 mm^2^, acquisition matrix = 64 × 64. For each participant, 235 image volumes were obtained. High-resolution T1-weighted structural images were also acquired for co-registration, using the sagittal 3D magnetization prepared rapid gradient echo (MP-RAGE) sequence with the following parameters: 176 sagittal slices, TR = 1900 ms, TE = 3.44 ms, slice thickness = 1 mm, flip angle = 9°, inversion time = 900 ms, FOV = 256 × 256 mm^2^, acquisition matrix = 256 × 256.

### Data processing

### Image preprocessing

fMRI data were preprocessed using Data Processing Ȧ Analysis of Brain Imaging (DPABI, http://rfmri.org/DPABI) [65]. Briefly, functional images were slice timing corrected, realigned, co-registered to high-resolution structural images, normalized to the Montreal Neurological Institute (MNI) space, resampled to 3×3×3 mm^3^, and smoothed with a 6-mm full-width half-maximum (FWHM) Gaussian kernel. Additionally, 17 participants with excessive head movement (translation > 3 mm or rotation > 3 degree in any direction) were excluded (10 Met carriers and 7 Val homozygotes), resulting in the inclusion of 121 participants in the functional image analysis.

### Voxel-wise task-related activity analyses

Task fMRI images were first analyzed by general linear models (GLM) using SPM8 (Statistical Parametric Mapping, http://www.fil.ion.ucl.ac.uk/spm/software/spm8) at the individual level. Four conditions (fixation, 0-back, 1-back, and 2-back tasks) with onsets and durations were set up, and the realignment parameters were included in the model as confounding factors. Contrast images of interest were created by contrasting the 0-back condition with the 2-back condition. To identify core brain regions activated or deactivated, task-related activation and deactivation masks were separately generated by group-level one-sample t tests, with age, years of education and *APOE* ε4 status as covariates (uncorrected p < 0.001). Further full factorial analyses were conducted with the *COMT* genotype (Met allele carriers vs. Val homozygotes) and sex (male vs. female) as independent factors for brain activation and deactivation under the corresponding brain masks, and age, years of education and *APOE* ε4 status were also included as covariates. Monte Carlo simulation was used for multiple comparison corrections (p < 0.005 uncorrected, 10,000 iterations) to achieve a cluster-level false-positive rate of 0.05 [66]. The resulting clusters with significant correlations with 2-back task performance were referred to as seed regions in further functional connectivity analyses (6 mm spheres centered at the peak coordinates of the clusters).

### Task-based background functional connectivity analyses

For each subject, residual images containing background activity information for the 2-back WM task were generated using the above GLM models by regressing the experiment design, head motion effect and other nuisance covariates, which were thought to be related to the act of performing the cognitive task [34]. Based on the residual image, the bFC was calculated as a Pearson correlation between the averaged time series of each seed region (based on activation analyses as described in the previous section) and a voxel in the rest of the brain during the 2-back condition. Fisher’s r to z transformation was applied to normalize the original bFC maps, followed by a whole-brain z-score standardization to rescale the FC values and reduce the impact of many sources of nuisance variates (e.g., head motion) using the DPABI toolbox.

Next, full factorial analyses based on the standardized bFC map were conducted to determine areas in which FC significantly differed between the two *COMT* genotypes and between the two sexes, with age, years of education and *APOE* ε4 status as covariates. After multiple comparison corrections using the same method as mentioned above, the mean bFC values of the resulting clusters were extracted for subsequent correlation analyses.

### Statistical analyses

PLINK software was used to assess Hardy-Weinberg equilibrium. SPSS 20.0 was used to conduct two-way analysis of variance (ANOVA) with the *COMT* genotype and sex as grouping factors to examine demographic variables (age and years of education). Two-way analysis of covariance (ANCOVA) was conducted to examine performance on the neuropsychological tests and the n-back WM task, with genotype and sex as the grouping factors and age, years of education and *APOE* ε4 status as covariates. Partial correlations were obtained to examine the relationships between task performances and task-related activation and task-based bFC, with age, years of education, *APOE* ε4 status, activation of the seed region, and reaction time of the 2-back task as covariates. A statistical significance level of p < 0.05 was used in these analyses.

## Supplementary Material

Supplementary Figures

Supplementary Tables
