# Supplementary material for: Female-specific effects of the catechol-O-methyl transferase Val158Met gene polymorphism on working memory-related brain function
Source: Aging (Albany NY). 2020 Nov 22;12(23):23900–16. doi: 10.18632/aging.104059 (PMC7762470; doi:10.18632/aging.104059)
Supplement: Supplementary Figures [file aging-12-104059-s001.pdf]

SUPPLEMENTARY TABLES

Supplementary Table 1. Demographics and working memory performance in the imaging subgroup.

	Met allele carriers (N = 44)		Val homozygotes (N = 77)		*COMT* $F/\chi^2(p)^a$	Sex $F/\chi^2(p)^a$	*COMT* × Sex $F/\chi^2(p)^a$
	Male (N = 19)	Female (N = 25)	Male (N = 31)	Female (N = 46)			
Age, yrs	67.05±8.30	63.32±7.94	66.97±6.88	64.91±6.68	0.29(0.59)	4.33(0.04)	0.36(0.55)
Education, yrs	11.63±2.87	10.60±2.93	12.03±3.59	11.54±3.02	1.25(0.27)	1.60(0.21)	0.20(0.65)
APOE ε4 carriers	5(26%)	7(28%)	7(23%)	16(35%)	0.09(0.76)	1.01(0.32)	1.45(0.69)
MMSE,score	28.21±1.36	27.80±1.47	28.29±1.66	27.98±1.54	0.03(0.87)	1.05(0.31)	0.01(0.92)
DST, score							
Forward	7.83±1.15	7.56±1.56	7.45±1.34	7.43±1.47	2.19(0.14)	0.24(0.62)	0.19(0.66)
Backward	4.79±1.18	4.52±1.08	4.74±1.50	4.70±1.35	< 0.01(0.99)	0.15(0.70)	0.13(0.72)
2-back task performance							
Reaction Time, ms	591.95±95.44	619.44±123.27	637.11±107.37	608.85±113.59	0.70(0.40)	0.02(0.89)	1.72(0.19)
Accurate Rate	0.93±0.05	0.92±0.05	0.92±0.05	0.89±0.10	2.48(0.12)	5.56(0.02)	0.07(0.80)

Values are means ± SD.
COMT, catechol-*O*-methyltransferase Val[158]Met genotype; MMSE, Mini-Mental State Examination; DST, Digit Span Subtest.
[a] Comparisons between groups were performed using Wald χ^2-test for *APOE* ε4. Two-way analysis of covariance (two-way ANCOVA) was used to determine the main effect of the genotype and sex on the MMSE and working memory performance, as well as the interaction between them (age, education, and *APOE* ε4 as covariates).

Supplementary Table 2. Effects of *COMT* genotype and sex effects on task-related brain activity.

	Brain region	MNI coordinates X	MNI coordinates Y	MNI coordinates Z	Cluster size	T value	P value
Activation regions	**Genotype effect: Met allele carriers > Val homozygotes**						
	Left inferior temporal gyrus	-54	-54	-12	68	3.84	1.01E-04
	Triangular part of left inferior frontal gyrus	-57	18	3	156	4.33	1.63E-05
	**Sex effect: Male > Female**						
	Left angular gyrus	-45	-69	39	246	4.22	2.45E-05
Deactivation regions	**Interaction effect:**						
	Right precuneus	3	-51	24	30	3.30	6.50E-04
	Right superior occipital gyrus	15	-87	27	37	3.67	1.87E-04

Supplementary Table 3. Effects of *COMT* genotype and sex on task-based background functional connectivity.

ROI seed	Brain region	MNI coordinates			Cluster size	T value	P value
		X	Y	Z			
Triangular part of left inferior frontal gyrus (Genotype effect seed)	**Sex effect: Male < Female**						
	Left calcarine	-9	-63	9	181	-4.10	3.82E-05
	Right calcarine	18	-60	12	56	-3.89	8.55E-05
	Left superior temporal gyrus	-63	-18	9	38	-3.89	8.53E-05
	Left rolandic operculum	-36	-30	15	25	-4.76	2.86E-06
Left angular gyrus (Sex effect seed)	**Genotype effect: Met allele carriers < Val homozygotes**						
	Right angular gyrus	45	-63	45	31	-3.37	5.09E-04
	**Sex effect: Male < Female**						
	Left medial superior frontal gyrus	-3	51	21	45	-3.94	6.96E-05
	Right superior temporal gyrus	57	-48	21	31	-4.27	2.00E-05
