# Supplementary material for: Female-specific effects of the catechol-O-methyl transferase Val158Met gene polymorphism on working memory-related brain function
Source: Aging (Albany NY). 2020 Nov 22;12(23):23900–16. doi: 10.18632/aging.104059 (PMC7762470; doi:10.18632/aging.104059)
Supplement: Supplementary Tables [file aging-12-104059-s002.pdf]

SUPPLEMENTARY FIGURES

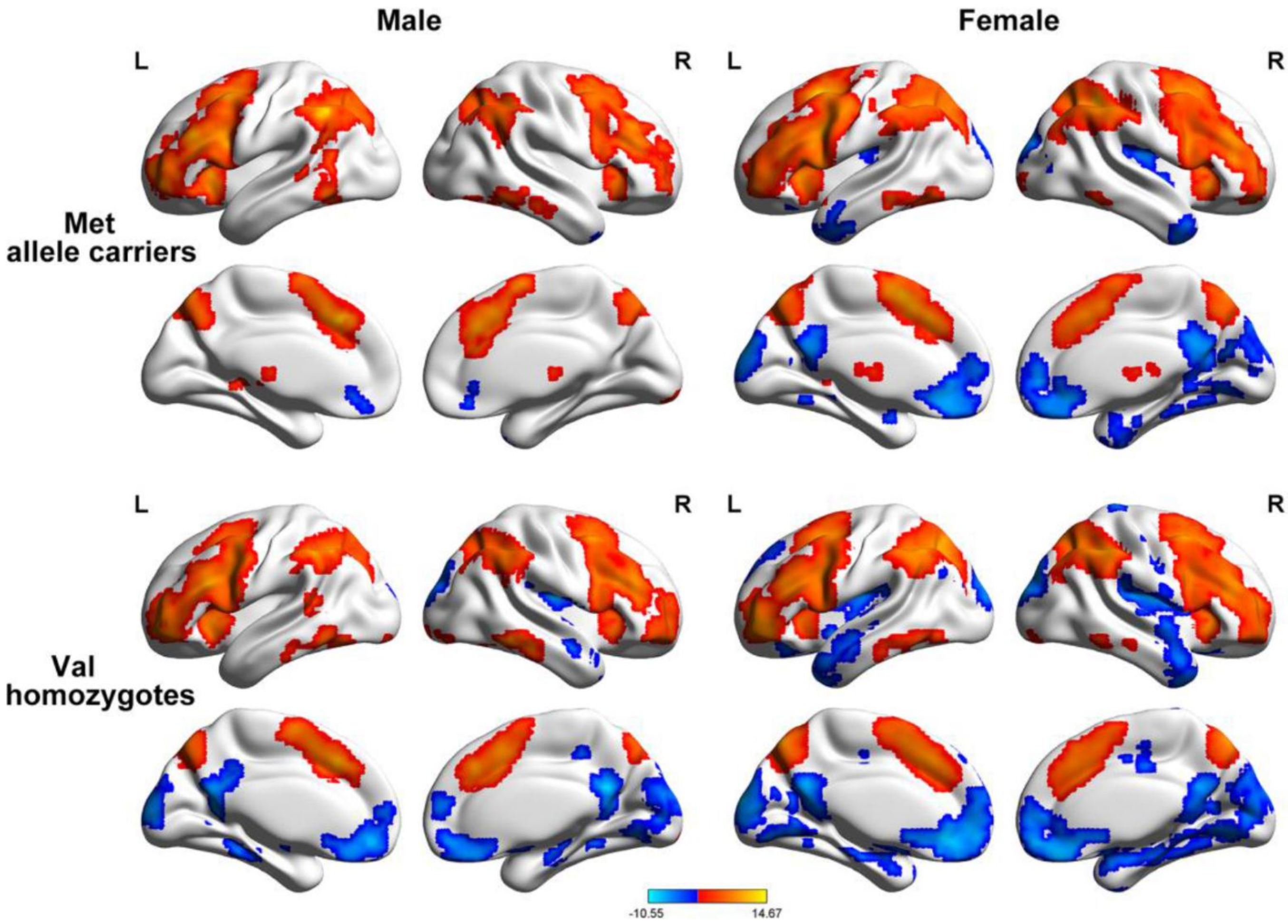

Supplementary Figure 1. The spatial maps show the brain activation and deactivation under working memory task for the genotype and sex groups separately. Group-level one-sample t test (age, years of education and *APOE* ε4 status as covariates) was conducted to generate task-related brain activation and deactivation for each group, with a threshold of voxel $p < 0.001$. Warm color, activation regions; Cool color, deactivation regions.

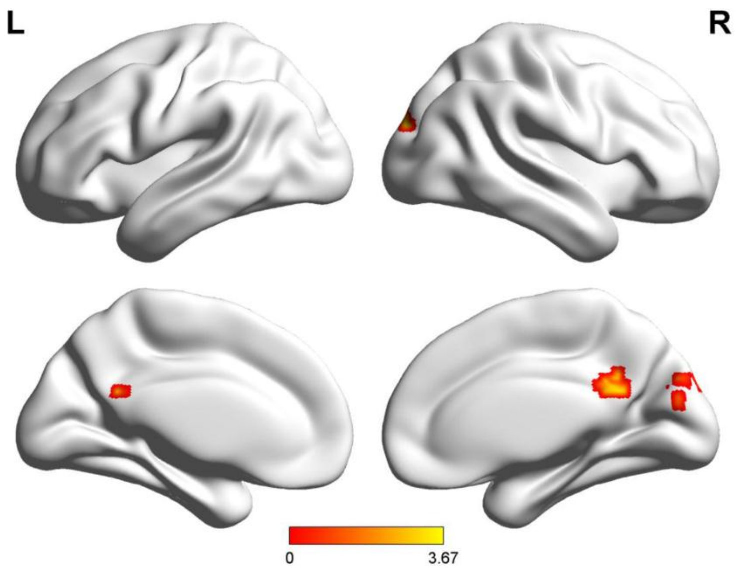

Supplementary Figure 2. Rendering of regions with significant interaction effect on task-related brain deactivation. Full factorial analysis with the *COMT* genotype (Met allele carriers vs. Val homozygotes) and sex (male vs. female) as independent factors was conducted for task-related brain deactivation regions, significant interaction effects were found in right precuneus and right superior occipital gyrus. Threshold, voxel $p < 0.005$ and cluster-level false-positive rate $p < 0.05$.

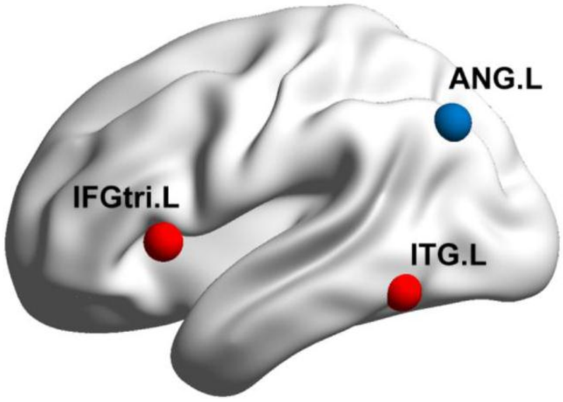

Supplementary Figure 3. Representation of the seed regions. Warm color, *COMT* genotype effect seeds; Cool color, sex effect seed; IFGtri.L, triangular part of left inferior frontal gyrus; ITG.L, left inferior temporal gyrus; ANG.L, left angular gyrus.
